# Therapeutic Notes

**Published:** 1896-02-22

**Authors:** 


### THERAPEUTIC NOTES.

### THYREO-ANTITOXIN.

### The Supposed Active Principle of Thyroid Extract.

Owing to the interest which has lately centred in the treatment of myxœdemic conditions and constitutional obesity by the exhibition of thyroid extract, it was inevitable that attempts to elucidate its chemical nature and isolate the active principle should follow. It was legitimate, on physiological grounds, to conclude that the train of symptoms which followed its administration were due to some ferment-like body or substance of albuminoid nature analogous to others recognised in the domain of sero-therapeutics. That this is not the case must be admitted if the following results, obtained by Dr. Sigmund Fraenkel, can be relied upon. In the published account of his researches his method was as follows. To an aqueous extract of the gland acetic acid was added to effect a precipitation of the albumin. The precipitate and the filtrate were severally tested as to their therapeutic action. The precipitate gave negative results; the administration of the filtrate was followed by the usual results attendant on ingestion of thyroid juice. It was obvious, then, that to separate the active principle attention had to be directed to the non-albuminous portion of the gland extract. A fresh portion of the extract was prepared and freed from albumin by the sugar-of-lead treatment; the residue was evaporated to a syrup and extracted with alcohol, and again precipitated with ether or acetone. The precipitate was a crystalline body, very hydroscopic, soluble in water and alcohol, slightly alkaline, and not precipitated by acetate of lead. Elementary analysis showed its formula to be $C_6H_{11}N_3O_5$. Its rational formula remains still to be discover ed, and until this is done it is useless to attempt its synthetic preparation. Thyreo-antitoxin, the name given by Dr. Fraenkel to the new body, is believed by him to be the true active principle of the ordinary extract—at least, so his physiological experiments lead him to believe. In the case of both animals and man, the train of symptoms following its administration are in entire harmony with this belief. Dr. Fraenkel's investigations require amplification and repeti tion; but, whether the real body has been discovered or not, additional light has been thrown on the chemistry of the subject.

